# A preclinical evaluation of alternative site for islet allotransplantation

**DOI:** 10.1371/journal.pone.0174505

**Published:** 2017-03-30

**Authors:** Chengshi Wang, Xiaojiong Du, Sirong He, Yujia Yuan, Pengfei Han, Dan Wang, Younan Chen, Jingping Liu, Bole Tian, Guang Yang, Shounan Yi, Fabao Gao, Zhihui Zhong, Hongxia Li, Jingqiu Cheng, Yanrong Lu

**Affiliations:** 1 Key Laboratory of Transplant Engineering and Immunology, Ministry of Health, Regenerative Medicine Research Center, West China Hospital, Sichuan University, Chengdu, China; 2 Department of Vascular Surgery, West China Hospital, Sichuan University, Chengdu, P R China; 3 Animal Center, West China Hospital, Sichuan University, Chengdu, China; 4 Center for Transplant and Renal Research, The Westmead Institute for Medical Research, Westmead Hospital, Westmead, New South Wales, Australia; 5 Molecular Imaging Center, Department of Radiology, West China Hospital, Sichuan University, Chengdu, China; 6 National Center for Safety Evaluation of Traditional Chinese Medicine, Chengdu, China; Children's Hospital Boston, UNITED STATES

## Abstract

The bone marrow cavity (BMC) has recently been identified as an alternative site to the liver for islet transplantation. This study aimed to compare the BMC with the liver as an islet allotransplantation site in diabetic monkeys. Diabetes was induced in Rhesus monkeys using streptozocin, and the monkeys were then divided into the following three groups: Group1 (islets transplanted in the liver with immunosuppressant), Group 2 (islets transplanted in the tibial BMC), and Group 3 (islets transplanted in the tibial BMC with immunosuppressant). The C-peptide and blood glucose levels were preoperatively measured. An intravenous glucose tolerance test (IVGTT) was conducted to assess graft function, and complete blood cell counts were performed to assess cell population changes. Cytokine expression was measured using an enzyme-linked immune sorbent assay (ELISA) and MILLIPLEX. Five monkeys in Group 3 exhibited a significantly increased insulin-independent time compared with the other groups (Group 1: 78.2 ± 19.0 days; Group 2: 58.8 ± 17.0 days; Group 3: 189.6 ± 26.2 days) and demonstrated increases in plasma C-peptide 4 months after transplantation. The infusion procedure was not associated with adverse effects. Functional islets in the BMC were observed 225 days after transplantation using the dithizone (DTZ) and insulin/glucagon stains. Our results showed that allogeneic islets transplanted in the BMC of diabetic Rhesus monkeys remained alive and functional for a longer time than those transplanted in the liver. This study was the first successful demonstration of allogeneic islet engraftment in the BMC of non-human primates (NHPs).

## Introduction

Allogeneic islet transplantation has been considered a potential treatment for type 1 diabetes mellitus (T1DM) [1],and islet and kidney co-transplantation could prolong the survival of kidney grafts, especially for TIDM patients with end-stage renal disease [2, 3].Various anatomical sites have been reported for islet transplantation, among which the liver has been primarily and widely used for clinical islet transplantation[4, 5]. However, several shortcomings of intrahepatic islet transplantation also have been reported, and approximately 50% to 75% of islet engraftments in the liver are lost after transplantation[6].

The instant blood-mediated inflammatory reaction is an innate immune response that causes significant islet graft loss[7–9]. Nevertheless, multiple factors lead to the loss of islet grafts, including high glucose levels, insufficient oxygen levels and high concentrations of drugs in the liver. The physiological and anatomical characteristics of the liver may also cause the death of transplanted islets, and technical obstacles have been associated with the addition of sufficient islets to the portal circulation[10–12]. The infusion of islets into the portal system also increased the portal pressure, which substantially reduces the viability of the transplanted islets.

Therefore, many studies have focused on developing alternative sites for islet transplantation. Perez et al. reported the long-term survival and function of allogeneic islet transplantation in the anterior chamber of the eye in diabetic baboons [13]. However, the anterior chamber in the human eye does not have sufficient space for islet transplantation to reverse hyperglycemia. Rafael et al. reported that autologous islets transplanted in the brachioradialis muscle released insulin for more than 2 years after transplantation in a young child [14]. Moreover, hypoxia has been demonstrated to be a key factor that leads to early islet loss, and the injection of a substantial quantity of islets has been demonstrated to cause massive early death in islet cells due to hypoxia. Therefore, the implantation technique has been argued to contribute to the high success rate at the intramuscular site. In addition, substantial fibrosis has been identified in this scenario in both experimental and clinical situations[15, 16].

Recently, the bone marrow cavity (BMC) has been suggested as an alternative site for islet transplantation[17, 18], and several advantages are associated with the use of the BMC as the site for islet allotransplantation. First, the transplantation of islets into the BMC is feasible and safe, and the surgical procedure is similar to that used for cord blood cell infusion in the BMC of patients with acute leukemia[19, 20]. Second, the islets will be well vascularized in the extra vascular environment of the BMC[17, 18]. Third, the BMC has been suggested to impart some degree of immunologic privilege[20–23], although the concept of immunologic privilege has recently been questioned [24, 25].Fourth, local secreted factors in the BMC, such as osteocalcin, epidermal growth factor receptor(EGFR), cAMP response element binding protein-1 (CREB1), myeloid cell leukemia-1 (MCL1) and vascular endothelial growth factor A (VEGFA), may prevent apoptosis and enhance islet graft survival and function[26]. Finally, islet cells in the BMC may stimulate cellular repair and regeneration and thus reduce procedure-related complications[17].

Non-human primates (NHPs) are an outstanding preclinical animal model because of their high genetic and immunological similarities to humans[27]. However, few reports have described islet allotransplantation in the BMC of NHPs. In this study, we aimed to investigate the survival and function of transplanted islets in the BMC and evaluate the suitability of the BMC (tibia) as an alternative site for islet transplantation in a preclinical animal model.

## Material and methods

### Animals and T1DM induction

Rhesus monkeys (3–5 years old, 5.8–6.9 kg in weight) were purchased from the Chengdu Ping'an Experimental Animal Reproduction Center (License No. SCXK (CHUAN) 2014–013, Chengdu, China). Diabetes was induced in the monkeys (fasting blood glucose (FBG) levels > 11.1 mmol/L on two consecutive days) with a single intravenous injection of streptozocin (STZ, 80 mg/kg, Sigma-Aldrich, CA, USA) 30 days prior to transplantation[28].

### Ethical statement

The animal protocols used in this work were approved by the Institutional Animal Care and Use Committee of the Traditional Chinese Medicine National Center (Chengdu, China) (Protocol:IACUC-2014001C), and the protocols strictly abided by the Laboratory Protocol of Animals created by the IACUC. The animals were housed at the Traditional Chinese Medicine National Center(Chengdu, China)in separate stainless steel cages (800×900×2060 mm) at a constant temperature (16–26°C) in HEPA-filtered air under a 12-h light and dark cycle from 7:30am-7:30pm and received insulin injections twice daily before feeding. The monkeys were provided opportunities to play with toys (polyvinyl chloride (PVC) toys and mirrors) while in their cages, and they could perch on the crossbars in the cages. The animal care staff and technicians talked softly to animals to provide animals with auditory stimulation and made good visual contact with the animals. The animals were fed a diet of monkey chow (Ke’ao Xieli Feed Co. Ltd., Beijing, China) twice daily and received sterilized water adlibium. None of the monkeys died during the experiment prior to euthanasia.

### Islet isolation and purification

The islets were purified and isolated as previously described [29, 30]. The pancreatic tissues were perfused with 40 ml collagenase P (1.5 mg/ml, Roche, Basel, Switzerland), and the perfused pancreatic tissues were cut into small pieces and digested at 37°C for 10 minutes with gentle shaking. The digestion was terminated by the addition of 200 ml cold Hank’s buffer containing 10% fetal calf serum. The islets were then collected by centrifugation and purified with human lymphocyte separation media (LSM; Sigma-Aldrich, St. Louis, MO).

### Islets labeled with SPIO

Super paramagnetic iron oxide (SPIO) was kindly provided by the College of Materials Science and Engineering of Sichuan University. A 5-μl dose of SPIO (200 μg/ml) was mixed with the islets in 5 ml of 1640 medium, and the mixture was cultured at 37°C for 2 hours. The islets were then washed and collected. For control purposes, the SPIO alone and islets alone were transplanted into the tibiae of monkeys.

### Allogeneic islet transplantation

After 16 hours of fasting, the recipient monkeys received an intramuscular (IM) injection of ketamine (10 mg/kg, Jiangsu Hengrui Medicine Co. Ltd., Jiangsu, China) and 20 m/kg IV propofol and fentanyl (Jiangsu Hengrui Medicine Co. Ltd., Jiangsu, China) to induce anesthesia. The animals were administered isoflurane and monitored using a breathing machine during islet transplantation. After surgery, the animals were injected with tramadol hydrochloride (100 mg i.m. every 12 hours) for analgesia for a total of 24 hours. The intrahepatic group(Group 1, n = 5) received an islet transplant into the liver as previously described [31]. Briefly, approximately 11,464 ± 2140 IEQ/kg of islets in combination with 200 U of heparin was slowly infused into the liver through the superior mesenteric vein. The monkeys were subsequently treated with immunosuppressive regimens. Animals that received BMC transplantation were divided into two groups: in Group 2 (n = 5), approximately 11,138 ±2,041 IEQ/kg of islets was transplanted into the tibial BMC without immunosuppressant. In Group 3 (n = 5), approximately 10,946±2,217 IEQ/kg of islets was transplanted in the tibial BMC with immunosuppressant. After transplantation, the blood glucose levels were assessed every morning using a Glucometer (Accu-chek Active, Roche, Basel, Switzerland). The first sign of graft function loss was 3 consecutive days of FBG >11.1mM, at which point the monkeys were treated with exogenous insulin. A fasting blood glucose level was>15mM under half the dose of exogenous insulin was considered a loss of graft function.

### Immunosuppressive regimen

Low-molecular-weight heparin was administered for anticoagulation; methylprednisolone served as an anti-inflammatory agent; anti-human thymocyte globulin (ATG), tacrolimus and sirolimus were administered to maintain immunosuppression (Sigma-Aldrich, CA, USA) (Table 1);and cefixime (Gerui Pharmaceutical Co., LTD, Guangdong, China) was used to prevent infection.

**Table 1:** Immunosuppressive regimen.

Treatment	Dose	Time Point	Route
**Low-molecular weight heparin**	300 IU/Day	Days -2,-1, and 0	i.m.
**Methylprednisolone**	250 mg	Day -1	i.m.
**Methylprednisolone**	25 mg	Day 0	i.m.
**ATG**	4 mg/kg	Days -1, 0, 1, and 2	i.v.
**Tacrolimus**	0.2 mg/kg/d	From day of Tx until graft failure	p.o.
**Sirolimus**	0.2 mg/kg/d	From day of Tx until graft failure	p.o.

i.m., intramuscular; i.v., intravenous; p.o., per os (by mouth); Tx, transplantation

### Evaluation of graft function

The graft function was evaluated by assessing the fasting blood glucose (FBG, every morning before feeding), fasting C-peptide (every 2 months), fasting insulin (every 2 months), exogenous insulin(every day depending on the FBG), and glycated hemoglobin (HbA1c, every 2 months) levels. The C-peptide and insulin levels in the bone marrow were measured every 2 months following islet transplantation. IVGTT was performed prior to STZ injection (normal group), after STZ injection(hyperglycemia group), and 1, 2, 4, and 6 months after transplantation. Briefly, after 16 hours of fasting, the monkeys were intravenously administered glucose (0.5 g/kg). Blood specimens were collected 0, 1, 3, 5, 10, 30, 60, 90 and 120 minutes after glucose injection. The glucose levels were measured in all blood specimens obtained. The serum was collected and analyzed for insulin using an electrochemiluminescence immunoassay (Roche Diagnostics, Basel, Switzerland). One month after transplantation, the glucose levels (0, 1, 3, 5, and 10 minutes)were measured in the bone marrow when IVGTT was performed.

### In vivo tracing of islets after transplantation

MRI scans of the tibia and the abdomen were performed 1, 30, and 225 days after transplantation on a clinical 3.0 T system (Sigma, General Electric, USA).The resultant images were quantitatively analyzed by manually counting the area of hypo-intense spots visible in the bone marrow tissue in each slice.

### Blood count and biochemical measurements

To assess the effect of islet infusion in the BM on the hematopoietic activity, aperipheral whole blood cell count was performed every 2 months after transplantation(Department of Experimental Medicine, West China Hospital).Kidney function and liver function were assessed using blood biochemical assays every 2 months after transplantation(Department of Experimental Medicine, West China Hospital).

### Serum collection

Blood was collected from all monkeys on the day of the transplantation and 10 days, 1 month, and 3 months after transplantation. The serum was immediately separated and maintained at -80°Cfor future use.

### ELISA

The levels of high-sensitivity C-reactive protein (Hs-CRP), thrombin-antithrombin complex(TAT)and fibrinogen (Fbg) in the serum were measured using a monkey enzyme-linked immune sorbent assay (ELISA) kit (Cusabio Biotech Co.,LTD, Hubei, China). The human soluble intercellular adhesion molecule-1 (sICAM) level was measured using an ELISA kit (Invitrogen, CA, USA) following the manufacturer’s instructions. All data were measured using a plate reader (Molecular Devices).

### MILLIPLEX

The MILLIPLEX MAP NHP cytokine kit was purchased from Merck Millipore (Merck Millipore, BOSTON, USA). The kit contained specific components for the assessment of NHP IFN-γ, IL-2,IL-10 and TGF-β. The assays were performed according to the manufacturer’s instructions, and the samples were measured on a Luminex 200 with the Milliplex Analyst Software.

### Immunofluorescence

Monkeys in Group 3 (at the first sign of a loss of graft function) were euthanized with an overdose of pentobarbitone sodium (iv, 300mg/kg) 225 days after transplantation. The bone marrow was collected and stained with DTZ and Prussian blue. The tibia containing islet grafts was removed and fixed in 10% formalin, decalcified in 10% EDTA for 30 days at room temperature and then embedded in paraffin. For the immunofluorescent staining, 5-μm sections were stained with rabbit-anti-human insulin polyclonal antibody (1:100; LifeSpan BioSciences, USA) and goat-anti-human glucagon polyclonal antibody (1:500; Santa Cruz Biotechnology Inc., USA). The secondary antibodies included goat-anti-rabbit FITC (1:500) and rabbit anti-goat Cy3 (1:500)(Golden Bridge Biological Technology, Beijing, China). The nuclei were stained with DAPI(Golden Bridge Biological Technology, Beijing, China).

### Flow cytometry analysis

Peripheral blood and bone marrow from monkeys were collected 10 days after islet transplantation. The mononuclear fractions of the peripheral blood and bone marrow were isolated using LSM. The cells were stained with monoclonal antibodies (mAbs), including FITC-conjugatedanti-CD4,PE-conjugatedanti-CD25, APC-conjugated anti-Foxp3 (eBioscience, San Diego, USA),PE-Cy5-conjugated anti-CD3 and PE-conjugated anti-CD8 (Beckman-Coulter, Miami, FL)according to the manufacturer’s instructions.

### Statistical analysis

All data are represented as the means ± standard deviations. One- or two-way ANOVAs followed by the t-test were used to assess the significance of differences using the SPSS software. For all analyses, a 2-tailed P value of 0.05 was considered significant.

## Results

### Islet function post-transplantation in monkeys

**Fig 1:**
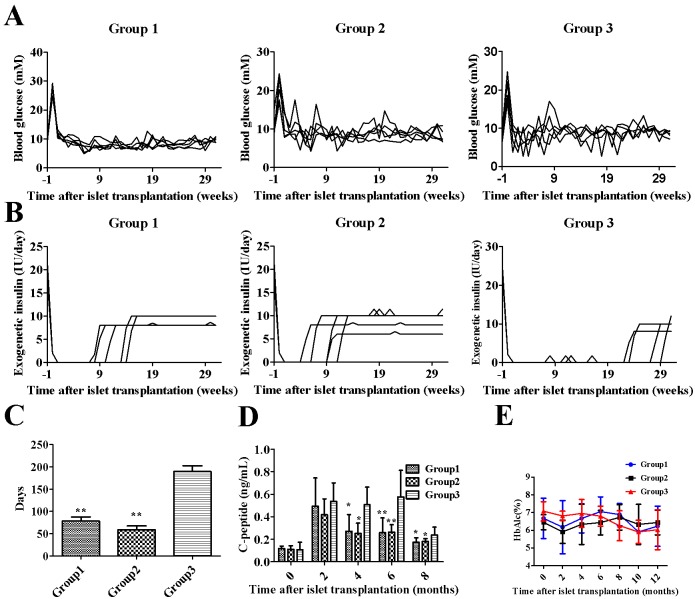
Metabolic control and graft function following islet transplantation. (A) Average FBG of diabetic monkeys following islet transplantation in each group. (B) Average exogenous insulin requirement expressed as IU/day. (C) Days of normal blood glucose levels without exogenous insulin. (D) Fasting serum C-peptide levels following islet transplantation. (E) HbA1c levels following transplantation. The data are expressed as the mean±SD. **, *P*< 0.01 versus Group 3; *, *P*<0.05 versus Group 3.

All blood glucose levels in the recipient monkeys decreased after transplantation (Fig 1A). The exogenous insulin dose depended on the blood glucose level of the recipient (Fig 1B), and Group 3 exhibited a significantly longer insulin-independent period than the other groups (Fig 1C).The serum C-peptide levels in Groups 1, 2 and 3 were significantly increased at months 2, 4 and 6(Fig 1D), and the serum C-peptide levels in Group 3 were significantly higher than those in the other groups at 4,6 and 8months. However, the glycated hemoglobin levels did not significantly differ among the three groups (Fig 1E). Islets transplanted into the BMC could reverse hyperglycemia and exerted better glycemic control than those transplanted in the liver.

**Fig 2:**
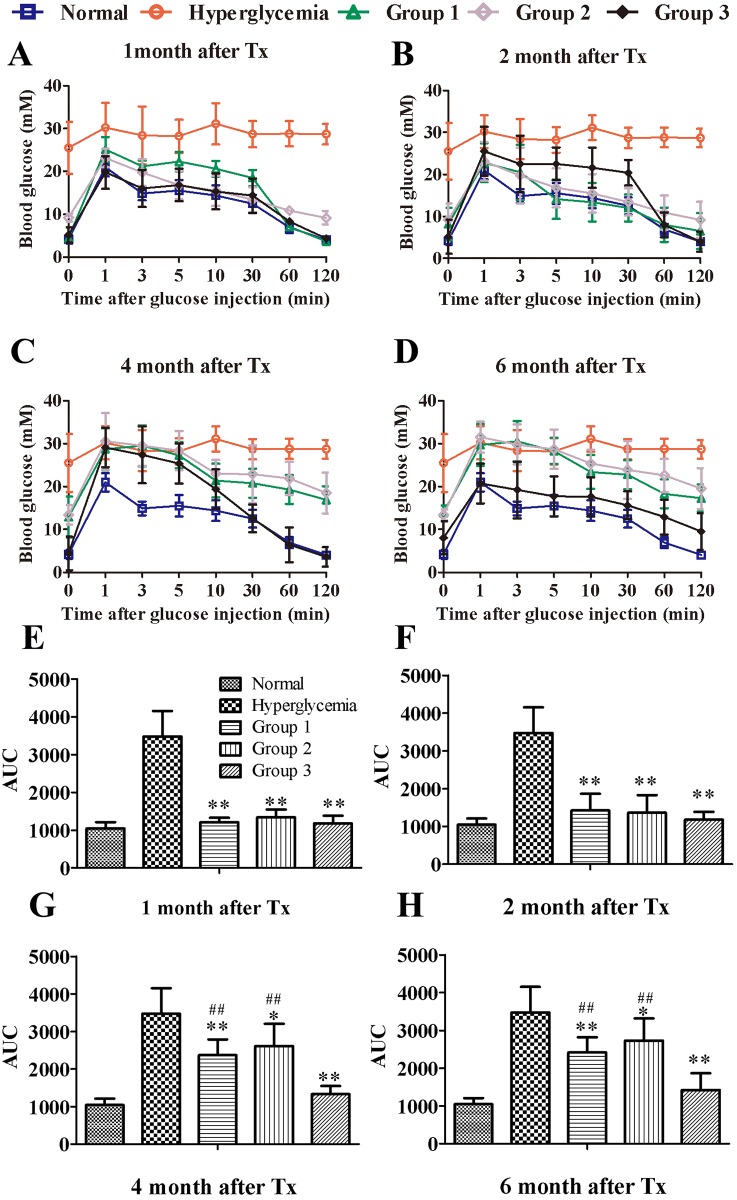
Blood glucose levels and AUC for glucose during IVGTT following islet transplantation in BM and liver. The IVGTT was measured 1, 2, 4, and 6 months after islet transplantation into the BM or liver. Normal and hyperglycemia were used as controls. (A-D) Blood glucose levels during IVGTT. (E-H) AUC for glucose during IVGTT. The data were expressed as the mean±SD. **, *P* < 0.01 versus the hyperglycemia group.*, *P* < 0.05 versus the hyperglycemia group. ##, P<0.01 versus Group 3.

The blood glucose changes in the IVGTT of the transplanted groups (Groups 1, 2 and 3) were similar to that of the normal group (Fig 2A–2D). The areas under the curve (AUCs) of the hyperglycemia group were significantly larger than those of the other groups 1, 2, 4, and 6months after transplantation. No differences were observed among the transplanted groups 1 and2 months after transplantation (Fig 2E–2H). The AUCs of Group 3 were significantly lower than those of Group1 and Group2 at 4 and 6 months after transplantation (Fig 2G–2H).

**Fig 3:**
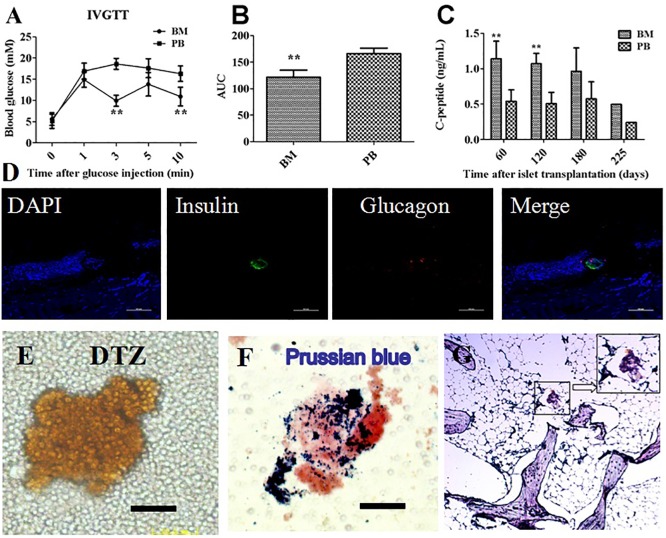
The identification of islets in the bone marrow cavity. Comparison of blood glucose levels (Fig 3A) and AUC for glucose (Fig 3B) in the peripheral blood (PB) and bone marrow (BM) during IVGTT following islet transplantation in Group 3. The C-peptide level (Fig 3C) was measured in the bone marrow and peripheral blood. Tibial bone was harvested 225 days after transplantation and stained for insulin and glucagon to confirm islet survival. The bone marrow collected from the tibia 225 days after islet transplantation was stained with DTZ(Fig 3E) and Prussian blue (Fig 3F). Tibial bone was stained for insulin and Prussian blue to confirm SPIO (Fig 3G).**, *P* < 0.01 versus PB group.

The glucose level in the peripheral blood of Group3 was higher than that of the bone marrow 3 and10 minutes after glucose injection (Fig 3A). The AUC of the IVGTT in the peripheral blood of Group3 was also significantly larger than that of the bone marrow (Fig 3B). However, there were no significant differences in the AUC of the IVGTT between the peripheral blood and bone marrow in Group1 and the normal group (S1 Fig).Moreover, the C-peptide levels of the bone marrow in Group 3 were significantly higher than that of the peripheral blood 60 and 120 days after transplantation (Fig 3C).

The immunofluorescence staining of tibiae revealed the presence of insulin and glucagon-positive cells at the site of transplantation (Fig 3D). We observed positive DTZ stains in the bone marrow from one monkey in Group 3 225 days after transplantation (Fig 3E). At the same time point, the Prussian blue stain of the bone marrow showed islets that were still labeled with SPIO (Fig 3F), and a histological analysis showed insulin and Prussian blue double-positive cells distributed in the area of the tibia injected with SPIO-labeled islets (Fig 3G).

### MRI of SPIO labeled islets in vivo

**Fig 4:**
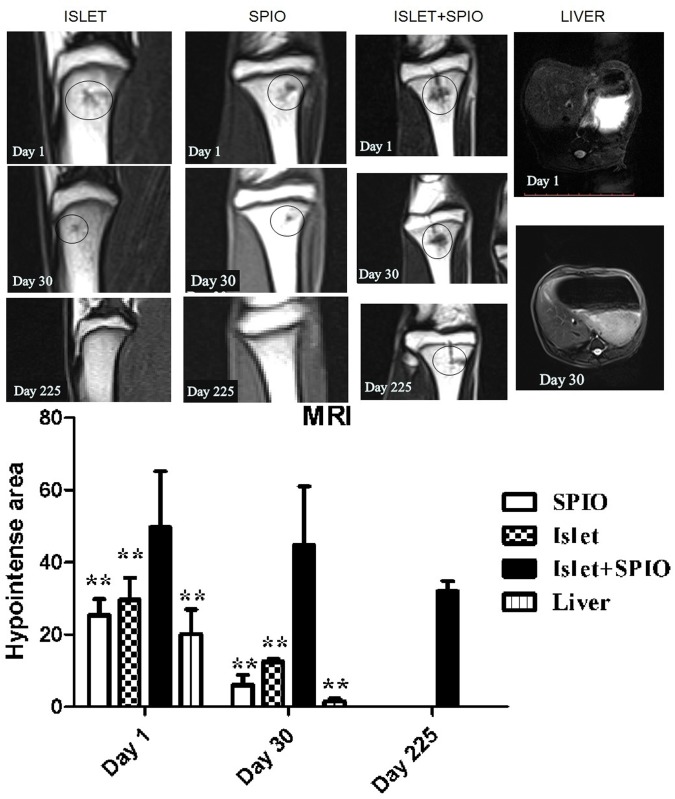
MRI of SPIO labeled islets in vivo. MRI was performed 1, 30 and 225 days after islet(left), SPIO (left middle) and islet+SPIO (right middle) transplantation. MRIs of the liver were acquired 1and 30 days after islet+SPIO (right) transplantation. A small hypo-intense area (black circle) inside the normal hyperintense signal was evident at the site of the islet infusion at the level of the tibia. The area of hypointense spots visible in the bone marrow tissue and liver in each slice in different groups (below). **, *P* < 0.01 versus islet+SPIO group.

Animals underwent MRI scans 1, 30, and 225 days after islet, SPIO, or islet+SPIO transplantation. As shown in Fig 4, the MRI results showed the hypointense signal at the islet, SPIO, and islet+SPIO infusion sites. Moreover, the area of hypo-intense spots was significantly larger in the bone marrow tissue islet +SPIO group than in the other groups at day 1 and day30 after transplantation (P<0.01, Fig 4 below). In addition, the hypo-intense area in the islet + SPIO group was also observed 225 days after transplantation, whereas SPIO-labeled islets that were transplanted in the liver were not observed 30 days after transplantation.

### Blood count and biochemical measurements

**Fig 5:**
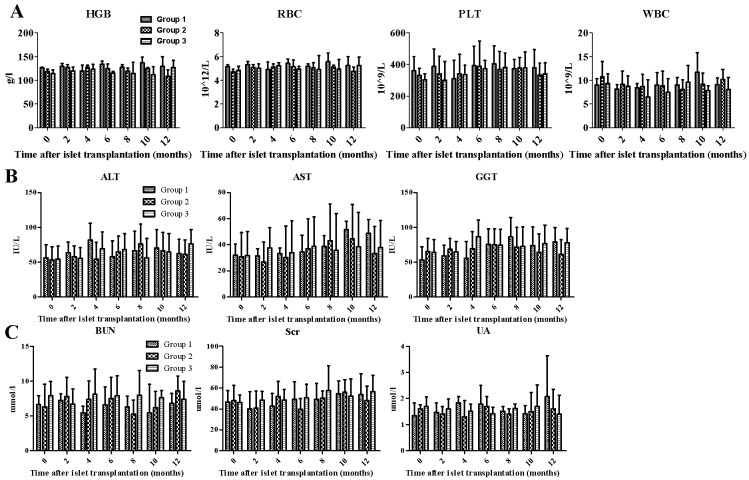
Blood count and biochemical measurements. The hematopoietic activity of the BM (HGB, RBC, PLT, WBC; Figure 5A), Liver function (ALT, AST, GGT; Figure 5B)and the kidney function (BUN, Scr, UA; Figure 5C) were measured every 2 months following transplantation. The data are presented as the means ± SD. A statistical analysis was performed using two way ANOVAs.

The peripheral blood was measured every 2 months after transplantation and did not show significant differences in the hematopoietic parameters, including the platelet count (PLT), hemoglobin(HGB),red blood cell, and white blood cell counts, among the three groups (Fig 5A). These findings demonstrated that the presence of the infused islets did not affect the hematopoietic activity of the BM during the 12-month period of observation. Moreover, the blood biochemical parameters, including liver functionality markers (i.e., Alanine aminotransferase (ALT),Glutamic oxalacetic transaminase (AST) and Gamma-glutamyl transpeptidase (GGT)) and kidney functionality markers (i.e., Blood Urea Nitrogen (BUN),Serum creatinine (Scr) and Blood uric acid(UA) were not significantly different one year after islet transplantation(Fig 5B and 5C).

### Serum cytokine analysis

**Fig 6:**
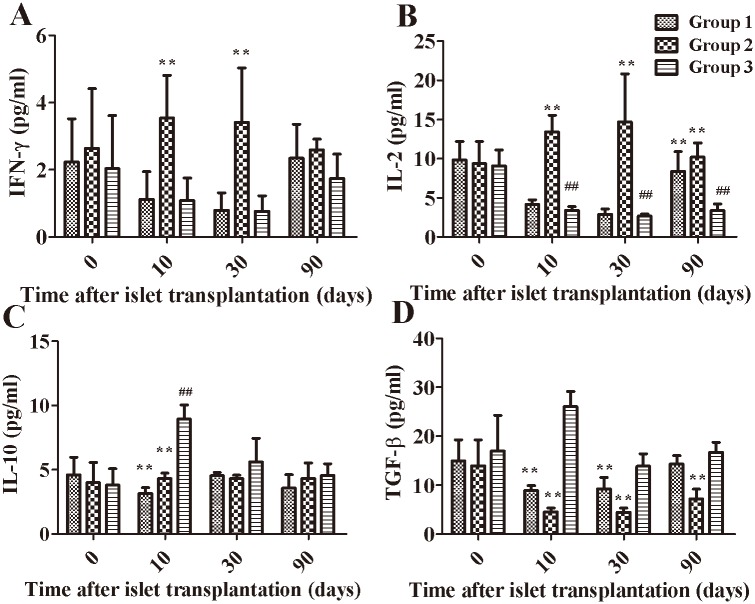
Serum cytokine levels in each group. The serum IFN-γ, IL-2, IL-10 and TGF-β levels were measured following islet transplantation. The data are presented as the means ± SEM. **, *P*< 0.01 versus Group 3; ##, *P*< 0.05 versus day 0.

The serum samples from each group were collected on 0, 10, 30, and 90 days after transplantation, and the resultant data are shown in Fig 6.The serum IFN-γ level was significantly lower in Group 3 than that in Group 2 on days 10 and 30 (Fig 6A). Moreover, the serum IL-2 level was significantly decreased in Group 3compared with Group 2 on days 10, 30 and 90(*P*<0.01). The serum IL-2 level was significantly decreased in Group 3 on days 10, 30 and 90 compared with day0 (*P*<0.01), and the serum IL-2 levels in Group 3 on day90 were significantly decreased compared with Group 1 (*P*<0.01) (Fig 6B). Furthermore, the serum IL-10 level in Group 3 on day 10 was significantly increased compared with day 0 (*P*<0.01), and the serum IL-10 level in Group 3 was significantly increased compared with the other two groups on day 10 (*P*<0.01) (Fig 6C). The serum TGF-β level in Group 3 was significantly increased compared with Groups 1and 2 on day10 (*P*<0.01). (Fig 6D). However, the TAT(thrombin antithrombin complex), sICAM, HS-CRP or Fibrinogen(Fbg) levels did not significantly differ between groups (data not shown).

### Bone marrow and peripheral blood lymphocyte subset distribution

**Fig 7:**
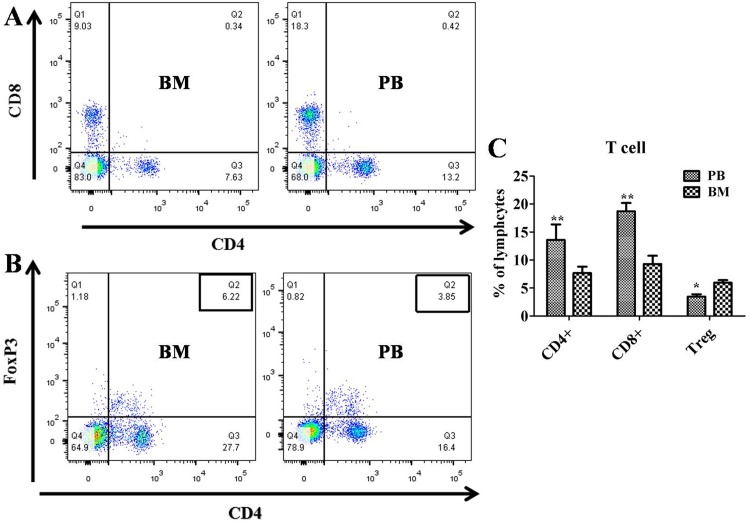
Bone Marrow (BM) and Peripheral Blood (PB) lymphocyte subset distributions. Bone marrow and peripheral blood were collected from Group 3 10 days after transplantation. (A) CD4+ and CD8+ T cells and (B) Treg cells were analyzed. (C) Percentage of lymphocyte subsets. Data are presented as the means ± SEM. **, *P*< 0.01 versus bone marrow group; *, *P* < 0.05 versus bone marrow group.

The expression levels of CD3+CD4+ and CD3+CD8+ were significantly lower in bone marrow cells than in the peripheral blood (both *P*<0.01, Fig 7A and 7C), whereas the expression of Treg (CD4+ FoxP3+) cells was significantly higher in the bone marrow than in the peripheral blood (*P*<0.05, Fig 7B and 7C).

## Discussion

Currently, the liver is the preferred receptor site for clinical islet transplantation, but this approach is associated with a number of important limitations. The purpose of our study was to investigate the BMC as an alternative site for islet allotransplantation in a preclinical model. We are the first group to demonstrate that transplanting islets in the BMCs of diabetic NHPs reversed diabetes.

Specifically, our results showed that islets could be easily and safely transplanted into the BMC, and no side effects were observed in Rhesus monkeys that had undergone BMC islet transplantation. Furthermore, none of the monkeys that had received a BMC transplant developed hypoglycemia after transplantation, and routine whole blood or blood biochemical indices of liver functionality (ALT, AST and GGT) and kidney functionality(BUN, Scr and UA)did not significantly differ between groups until one year after islet transplantation. Transplantation into the BMC is considered a less invasive and low-risk ambulatory procedure in clinical settings that provides unlimited opportunities for repeated transplantation and an easily accessible site for engraftment[26]. Frassoni et al. identified no side effects after the patients received a 20-ml infusion of a cell suspension[32], and Cantarelli E et al. reported that the appearance of islets did not affect the cellularity, cell subpopulation ratios or progenitor cell numbers in the BMC [20]. In contrast, a long-term follow-up study reported an increased risk of adenomas and hepatocellular carcinoma associated with intraportal islet transplantation[33].Surprisingly, our study did not identify side effects on hematopoietic cell function, whereas other groups reported a limited effect on bone density at the BM site used for transplantation[20].

Moreover, islets engrafted in the BMC exhibited better function than those transplanted in the liver. Specifically, our results demonstrated that islets transplanted to the BMC remained insulin-independent longer than intrahepatic transplanted islets(189.6 ± 26.2 days vs. 78.2±19.0 days). The IVGTT result also confirmed that the islet graft was functioning well in the BMC. Kover et al. reported that isograft islets transplanted in the BMC of dark agouti rats lasted longer (10–14 days) than islets transplanted into the liver and kidney capsule (3 days) [17, 26]. Cantarelli E et al demonstrated that only a small amount of islet isografts was required in the BMC of diabetic mice, and 76% of the mice transplanted with a marginal number of islets (250 IEQ) were insulin independent for at least 14 days [20].

Changes in the graft were more easily monitored in the BMC than in the liver. After the islets were transplanted into the liver via the hepatic portal vein, the islets were immediately randomly scattered, which may have precluded the detection of hypo-intense spots 30 days after transplantation into the liver. Therefore, harvesting biopsy samples of the islets to monitor early graft changes was difficult in the liver transplantation group, and Toso C et al. reported that less than30%of biopsies contained the desired islets [34]. In contrast, islets were easily harvested from the BMC because the BMC is a well-confined site and permits repeated biopsies.MRI also confirmed that the BMC was a better site for monitoring than the liver.

In this study, bone marrow samples were collected 10 days after transplantation, and flow cytometry was performed to measure the changes in T cell (CD3, CD4 and CD8) and Treg populations. CD3+CD4+ T cells are important in the initiation of the immune response because they recruit macrophages[35, 36]. For example, Yin D. P. et al. demonstrated that, in the absence of CD4+ T cells, activated CD8+ T cells may cause the acute rejection of xenotransplanted cellular grafts [37].Furthermore, Goto et al. demonstrated that CD3+CD4+ and CD3+CD8+ T cells as well as macrophages infiltrated the islet xenograft, which indicated that the rejection mechanisms in the bone marrow were similar to those described in a previous study [38]. In our study, the long-term survival of grafts in Group 3 maybe due to the lower levels of CD3+CD4+ andCD3+CD8+ T cells and higher levels of Treg cells around the grafts in the BMC. Although precisely identifying the types of cells that contribute to immunological tolerance is difficult, our results showed that islet grafts survived for long periods in Group 3. Our data indicated that the lower levels of CD3+CD4+ and CD3+CD8+ T cells and the higher levels of Treg cells and other progenitor cells around the grafts in the BMC, such as MSCs[39–42] and DCs [43, 44],may be involved in the survival of grafts. Fiorina P et al also demonstrated that hematopoietic stem cells promote the long-term survival of islet allografts via a PD-L1-mediated mechanism[45].

To better elucidate the mechanisms involved in graft survival, we also analyzed growth factors (IL-2, IL-10, TGF-β, and IFN-γ) secreted by cells in the BMC because other factors secreted in the BMC, such as osteocalcin, EGFR, and VEGFA, have been reported to enhance the survival and function of islet grafts[26]. Our results showed that the serum IL-10 and TGF-β levels in the immunosuppressed group that had received BMC transplants were higher than those in the other groups. IL-10 is a well-documented immunosuppressive cytokine that suppresses T cell activation, prevents the antigen-presenting process, and inhibits pro-inflammatory cytokines [46]. These findings indicate that the BMC may provide a suitable microenvironment to enhance long-term islet graft survival and function via cytokines secreted by stem cells or other cells in BMC. Other studies have also showed that the BMC could provide suitable oxygen tension, pH and nutrients for islet survival[18].

In conclusion, we demonstrated that islets were easily and safely transplanted and monitored in the BMC. Thus, the tibial BMC is an alternative site for clinical islet transplantation and cellular transplantation for other diseases.

## Supporting information

S1 Fig Blood glucose levels and AUC for glucose during IVGTT following islet transplantation in BM and PB. Comparison of blood glucose levels (S1A Fig) and AUC for glucose (S1B Fig) in the peripheral blood (PB) and bone marrow (BM) during IVGTT following islet transplantation in Group1, Group 3 and normal group. **, *P* < 0.01 versus PB group.
(TIF)
